# Writing with the Eyes: The Effect of Age on Eye-Tracking Performance in Non-Disabled Adults and a Comparison with Bimanual Typing

**DOI:** 10.1155/2021/9365199

**Published:** 2021-08-24

**Authors:** Marco Caligari, Marica Giardini, Ilaria Arcolin, Marco Godi, Stefano Corna, Roberto Colombo

**Affiliations:** ^1^Istituti Clinici Scientifici Maugeri, IRCCS, Scientific Institute of Pavia, Integrated Laboratory of Assistive Solutions and Translational Research (LISART), Pavia, Italy; ^2^Istituti Clinici Scientifici Maugeri, IRCCS, Scientific Institute of Veruno, Division of Physical Medicine and Rehabilitation, Gattico-Veruno, Italy; ^3^Istituti Clinici Scientifici Maugeri, IRCCS, Scientific Institute of Veruno, Service of Bioengineering, Gattico-Veruno, Italy

## Abstract

Eye-tracking technology is advancing rapidly, becoming cheaper and easier to use and more robust. This has fueled an increase in its implementation for Augmentative and Alternative Communication (AAC). Nowadays, Eye-Tracking Communication Devices (ETCDs) can be an effective aid for people with disabilities and communication problems. However, it is not clear what level of performance is attainable with these devices or how to optimize them for AAC use. The objective of this observational study was to provide data on non-disabled adults' performance with ETCD regarding (a) range of eye-typing ability in terms of speed and errors for different age groups and (b) relationship between ETCD performance and bimanual writing with a conventional PC keyboard and (c) to suggest a method for a correct implementation of ETCD for AAC. Sixty-seven healthy adult volunteers (aged 20–79 years) were asked to type a sample sentence using, first, a commercial ETCD and then a standard PC keyboard; we recorded the typing speed and error rate. We repeated the test 11 times in order to assess performance changes due to learning. Performances differed between young (20–39 years), middle-aged (40–59 years), and elderly (60–79 years) participants. Age had a negative impact on performance: as age increased, typing speed decreased and the error rate increased. There was a clear learning effect, i.e., repetition of the exercise produced an improvement of performance in all subjects. Bimanual and ETCD typing speed showed a linear relationship, with a Pearson's correlation coefficient of 0.73. The assessment of the effect of age on eye-typing performance can be useful to evaluate the effectiveness of man-machine interaction for use of ETCDs for AAC. Based on our findings, we outline a potential method (obviously requiring further verification) for the setup and tuning of ETCDs for AAC in people with disabilities and communication problems.

## 1. Introduction

Eye-tracking technologies, i.e., technologies that automatically track the point of an individual's gaze while that person views or interacts with a visual image, are widely used now in several research fields [1]. Since 1994, the field of language science has seen an exponential rise in the publication of eye-tracking research studies.

With this technology, the position of the eyes is determined based on light reflected from the curvature of the corneas and the position of the pupils. A low level of infrared light, which is invisible to humans, reflects off the eyes and a camera captures the images and computes the angle of reflection. A signal processing procedure matches the physical orientation of the eyes to specific *x*-*y* coordinates and maps them, through appropriate calibration, to the monitor's coordinate system. In this way, the point of gaze is sampled and converted into *x*-*y* coordinates.

The study of eye movement tracking has two main targets: (a) tracking for investigation (passive use) and (b) tracking for control (active use). The first refers to the use of eye-tracking technology in cognitive science, educational science, psycholinguistics, and experimental psychology to investigate matters such as cognitive load, processing of different channels, and attention distribution [2]. In particular, several studies have evidenced age-related differences in eye tracking. As people grow older, their saccade initiation, i.e., the command to the eyes to move from one point to another, gets slower [3], and there are significant differences in the patterns of visual search for information on text reading [4]. Moreover, with aging, certain cognitive capacities tend to decline. Some individuals can experience a mild cognitive impairment, which hinders the proper execution of some cognitive processes [5]. In general, the performance deficit of elderly people when using the technology is not due to a lack of experience or usability issues but to their cognitive conditions [6].

The second target, i.e., eye tracking for control, plays an important role in the development of Augmentative and Alternative Communication (AAC) technologies [1]. AAC is an effective means to supplement and enhance the functional communication skills of individuals with communication and language impairments [7, 8]. It can help them carry out daily activities when they cannot communicate through conventional speech.

In people with acquired neurological conditions, Eye-Tracking Communication Devices (ETCDs) have proven to be effective for the communication of essential needs to the caregiver and for the recovery of writing abilities and general communication issues [9, 10]. ETCDs enable users to produce text by using the focus of the gaze [11]. The principal reason for using eye typing is that eye movement muscles are often the last remaining functioning muscles in the presence of acquired neurological conditions. Hence, gaze input allows users to interact “hands free” with a personal computer and communicate with other people. This requires normal eye movements and vision as well the capability to hold the gaze for a short while (called “dwell-click time”) on the target (i.e., a letter or word), typically for a period of 200–600 ms [12].

The typing speed allowed by the eye trackers in combination with an on-screen keyboard has been the object of investigation since the early 90s. Gips and Olivieri [13] wondered if an eye typist could write at least one character per second (char/s) but, even if in theory this was possible, actually this speed is hardly ever reached [14]. Salvucci [15] developed advanced methods based on gaze fixation analysis to reconstruct the word entered by the user from the path and permanence of the gaze. The advantage of this was that it was not necessary to set a dwell-click time, but the system needed to have an internal dictionary to search, which became time consuming as the number of dictionary entries increased and failed when the user made a mistake. In addition, the authors found strong evidence that non-disabled users can improve their performance in the eye-based interface tasks, even after a small number of trials. Thus, repetition and its relation with age, which is known to influence the learning speed, can provide information about the duration and minimum number of learning trials necessary for the use of an ETCD.

Hansen et al. [16] studied the speed and accuracy of ETCD typing by using a particular on-screen keyboard with big-height buttons and a predictive function that redefined continuously the letters and words shown on each button. However, the performance with this new approach was influenced by the efficacy of the predictive algorithms. The typing speed was better than that obtained with previous methods (about 0.48 char/s), but the incidence of errors was high (about 28% overproduction). When using automatic dwell-click with eye-tracking AAC systems, the overproduction errors are typically due to a particular effect known as the “Midas touch” which consists of an involuntary activation of the active elements of the graphic interface (buttons and icons) caused by normal exploration of graphic elements through the gaze [16, 17].

Furthermore, the type of AAC software used may influence the typing performance. For example, Grid3 software (Smartbox Assistive Technology Ltd., Malvern, UK) and other modern eye-gaze enabled communication software include rest buttons to allow users to get familiar with the screen and avoid the Midas touch. Additionally, the dwell time and other aspects can be customized for the user.

To date, it is not entirely clear if the technological advances incorporated in the new ETCDs available on the market yield a superior performance in terms of greater writing speed with a reduced number of errors, such as to encourage users to adopt them for their communication. Hence, the provision of normal performance values (speed and accuracy) and their age distribution may be useful for setting up and optimizing ETCD systems for AAC and useful also as preliminary data for future testing to establish age norms. The aim of this study was (a) to evaluate the effect of age on speed and errors of ETCD performance in non-disabled adults using a commercial ETCD system; (b) to assess the relationship between ETCD performance and bimanual writing using a conventional PC keyboard; and (c) to outline a potential method, based on our findings in healthy people, for the setup and optimization of ETCDs for disabled people requiring AAC.

## 2. Materials and Methods

### 2.1. Participants

Healthy volunteers (*n* = 67, aged 20–79 years, 27 males and 40 females) were recruited at the Laboratory of Augmentative and Alternative Communication and Home Automation of the Istituti Clinici Scientifici Maugeri, IRCCS, Scientific Institute of Veruno (Italy). Inclusion criteria were as follows: no prior experience of ETCD use; no cognitive impairments, defined as ≤2 errors on the Short Portable Mental Status Questionnaire [18] (as a sign of the ability to understand the tasks administered); normal or corrected-to-normal vision (eyeglasses or contact lenses); ability to execute the 9-point ETCD calibration procedure; and capability to write by clicking with the fingers on a PC physical keyboard. Professional typists were excluded from the study. The study was carried out in conformity with the Declaration of Helsinki of the World Medical Association; all participants gave their informed consent to take part in the study, which had been approved by the institutional Scientific and Ethics committees (approval number: #680 CEC).

### 2.2. Eye-Tracking Communication Device

Participants were tested using the ETCD iAble-Mytobii© P10 (Tobii Technology, Danderyd, Sweden). It included a 15-inch LCD monitor (4/3 screen ratio), sample frequency of 40 Hz, and angular gaze detection accuracy of 0.5° (0.5 cm at 50 cm eye-screen distance). Participants were seated on a chair in front of the ETCD, which was mounted on a movable arm. The LCD screen was placed at 52 cm (∼2 cm) from the user's eyes and positioned so that the midline connecting both eyes was at the same height as the screen center. A headrest was used to keep the head position constant throughout the test. The MyTobii P10® ETCD worked in combination with the iAble© software ver. 1.2 (SRLabs Co., Milan, Italy) specifically designed for eye-tracking interaction. It features large screen items such as key buttons and icons to facilitate user interaction. In particular, each button of the iAble keyboard has an important feature: it shows separately the letters and the visual buttons that serve to write the relative letter (see Supplementary Material 1 for full details). In practice, the visual buttons (active part) are situated 1 cm to the right of each letter (passive part). Holding the gaze on the letter does not produce any text entry: the user needs to hold the gaze on the button corresponding to letter in order to activate entry. This strategy was adopted to reduce typing errors due to the “Midas touch” effect. In addition, the iAble keyboard gives a synthesized click sound as feedback to confirm each letter when it is typed, which allows the user to move their gaze and focus on the next one [19].

The full layout of the iAble virtual keyboard had an on-screen useful size of 12.3 × 18.7 cm and was located in the center of the LCD display (30.5 × 22.8 cm in size). In this way, each letter was displayed inside a rectangle of about 2.5 × 1.6 cm. The area above the virtual keyboard showed a text box (5 × 20 cm) to display the written text, while on the left and right sides there were some special command buttons to exit the application, cancel a message, or to activate speech synthesis of the written text. The iAble keyboard showed the letters arranged in a sequential alphabetic layout (ABC layout) consisting of 5 lines plus one for the space button. Unfortunately, this layout is different from the more diffused QWERTY layout, but it was the only one available for the device.

In accordance with Jacob et al.'s suggestion [12], the dwell-click time was set at 200 ms. We estimated that this could allow a good balance between typing speed and prevention of involuntary selection of wrong letters or command icons. The “word prediction” [20] and “automatic text completion” functions [21] were deactivated, in order to measure the raw “key striking rate” and not the maximum “typing speed” facilitated by different speed-up technologies, which could produce different performances with the different devices.

### 2.3. Eye-Tracking Typing Test

Before beginning the typing test, participants carried out the ETCD calibration procedure, during which we instructed them to maintain their gaze on a round target that was moved in different positions on the LCD screen. The device recorded the user's eye movements and fixations during this calibration procedure and calculated a reliability score. If the algorithm incorporated in the ETCD judged the score as fair, the participant proceeded on to the typing test; otherwise, the calibration procedure was repeated. After 3 consecutive unsuccessful calibration procedures, the participant was excluded from the study.

After a successful calibration, the examiner gave the participant the following instruction. “You have to hold your gaze until an acoustic feedback (click) informs you of the typed entry. Then sequentially move your gaze to each letter that composes the sample sentence “PROVA DI SCRITTURA” (i.e., “*writing test*”, in Italian). You must complete this task as quickly as possible. If you make an error, do not delete the entry or try to correct it but continue to type the sentence without skipping any letter.” Then, the command “START” was given to commence the eye-tracking typing test. When the user typed the last letter of the sentence, the command “STOP” was given and the trial ended. Each participant performed 11 consecutive test trials, with a 30 s resting period between each trial.

### 2.4. Manual Test with PC Keyboard

After the eye-tracking typing test, participants were requested to execute a bimanual writing test with a standard QWERTY physical PC keyboard. The keyboard was placed 5 cm from the edge of the desk, at a height of 65 cm from the floor and connected to a PC with LCD display. The instruction was “write the sample sentence with two hands and using as many fingers as you wish.” The test sentence was the same as that used in the previous test. As before, participants were asked not to cancel any erroneous entry but to continue by writing the correct letter and proceed typing until the end of the sentence.

### 2.5. Performance Measures

The writing time and the number of striking errors (i.e., wrong letters typed) were recorded during each trial, in both the ETCD and manual PC tests. The examiner measured the writing time of the sample sentence using a centesimal stopwatch and recorded it in an MS Excel worksheet. The measurement of typing time started at the emission of the first click, relative to the typing of the first letter of the 18-character sentence, and ended at the click corresponding to the typing of the last letter. Then, we computed the performance measures, namely, the typing speed, i.e., ratio between the time taken and the number of characters, in “characters per second” (char/s), and the error rate in percentage units [(number of err*∗*100)/number of total characters].

### 2.6. Statistical Analysis

General demographic data such as age, sex, dominant side, use of glasses/lenses, age, and education were collected before testing. We performed a basic statistical analysis on subjects' demographic data and performance values, expressing data as frequency or mean value (±standard deviation (SD)). We initially divided participants into 6 age groups (one for each decade) and performed a preliminary one-way ANOVA on ETCD typing speed with age group as the factor and Bonferroni test for pairwise comparisons. A significance level of <0.05 was set for all the statistical tests. Based on the preliminary analysis, we collapsed the 6 age groups into 3 broader categories (young, middle-aged, and elderly) and used these for the following analyses. Then, two-way repeated measures ANOVA with one between-subjects factor (age group) and one within-subjects factor (the repeated trials) was carried out on ETCD typing speed and error rate. In order to assess the differences between pairs of repeated sessions of each age group, one-way analysis and Bonferroni post hoc test were executed for each age group category and session pair. In addition, using least square exponential fitting modelling, we assessed on the same variables the learning curve (i.e., the non-linear increase/decay) typical for each independent group to evaluate the minimum number of trials required to achieve a stable performance. For this, we considered that the learning was concluded (steady-state value reached) after a time corresponding to 2 time constants of the exponential function, i.e., when 86.5% of the maximum/minimum asymptotic value was reached. The increasing model *y*(*x*)=*a* − *b∗e*^(−*x*/*c*) ^ and the decaying model  *y*(*x*)=*a*+*b∗e*^(−*x*/*c*) ^ were fitted, respectively, on typing speed and error rate data.

Finally, correlation analysis was performed to assess the relationship between ETCD and bimanual typing performance variables. Statistical analysis was performed using the STATA R13.0 statistical software package (StataCorp LLC, College Station, Texas, USA) and MATLAB custom software (2014b, MathWorks, Natick, MA, USA).

## 3. Results

Four (6.0%, 3 females, 1 male) out of the 67 subjects recruited were unable to successfully complete the ETCD calibration procedure and were excluded from the study. Demographic data according to age decade for the remaining 63 subjects and basic statistics are reported in Table 1. In the first four decades (age range: 20–60 years), the majority of subjects (60–90%) typed with a keyboard for work or pleasure on a daily basis, while the remaining used a keyboard only occasionally. Conversely, in the 60–69 year age group, most subjects (60%) keyboarded occasionally, while 20% keyboarded on a daily basis and the remaining 20% did not normally keyboard. A different behavior was found in the last decade, where 80% of subjects did not normally type on a keyboard in their daily life.

### 3.1. ETCD Typing Speed

**Figure 1:**
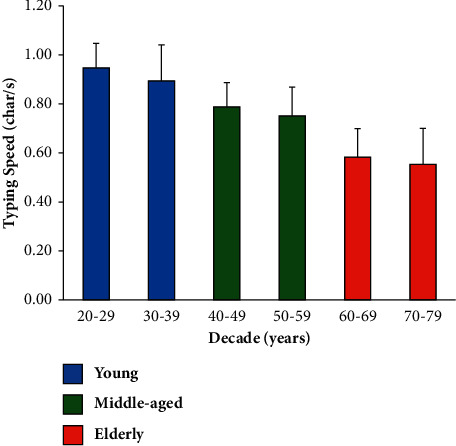
ETCD mean typing speed and standard deviation for each age decade of the participants who completed the study.

Figure 1 reports the mean ETCD typing speed for each age decade of the subjects who completed the study. As can be seen, typing speed decreased as age increased. Based on one-way ANOVA and Bonferroni test for all pairwise comparisons (see Supplementary Material 2 for full details), we collapsed the six decades into three age groups (young, middle-aged, and elderly). The new age groups are reported with the same color in Figure 1.

Table 2 reports the mean ETCD typing speed and error rate across the 11 measurement trials in the 3 age groups. The ETCD typing speed ranged from 0.43 to 0.99 char/s and increased progressively with repetition of the trials for each age group. The Bonferroni pairwise comparisons showed that the values of trial 1 were significantly different (*p* < 0.05) from those of the other trials starting from trial 5 for the young, from trial 4 for the middle-aged, and from trial 6 for the elderly.

**Figure 2:**
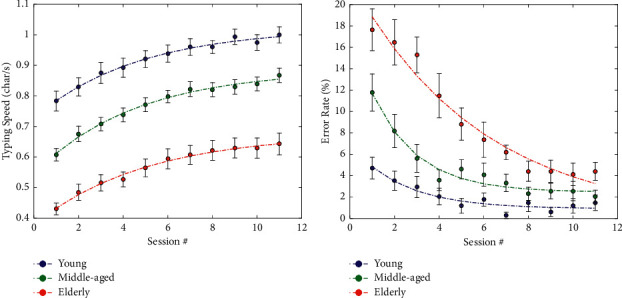
Non-linear models fitted to describe the learning effect due to repetition of the protocol. (a) Mean typing speed and (b) mean error rate across the 11 trials in the 3 age groups. Dashed lines represent the increasing/decaying exponential model fitted on the data. Error bars represent the standard error of the mean in each measurement trial and age group.

The two-way repeated measures ANOVA results showed a statistically significant effect (*p* < 0.001) both for age group and test trial. There was no significant interaction between the two factors. In other words, typing speed increased with a similar trend in the 3 age groups but with different levels of performance (see Figure 2(a)).

### 3.2. ETCD Error Rate

It ranged, for each subject, from 0% to 41%. The two-way repeated measures ANOVA showed that the error rate significantly (*p* < 0.001) increased with age and significantly (*p* < 0.001) decreased with the number of trial repetitions. There was a significant interaction between the two factors. This means that the trend of error rate decay differed among the 3 age groups (see Figure 2(b)).

### 3.3. Learning Model

Figure 2 shows the non-linear model we fit to describe the learning effect due to repeated trials. The exponential fitted model provided an excellent representation (see below) of the measured data for each age group and variable with a similar but distinct time course for typing speed and a different time course for error rate.

Table 3 reports the exponential model features both for typing speed and error rate in the three age groups. All these models could explain most of data variability with an adjusted *R*^2^ coefficient ranging from 0.86 to 0.99. The time constants of the typing speed were quite similar in the 3 age groups with the elderly showing the longest adaptation time (4.98 trials). The error rate decreased exponentially with a decaying time constant of about 2 trials in young and middle-aged participants and 5.84 trials in elderly subjects.

### 3.4. Relationship between ETCD Performance Variables and Bimanual Performance

**Figure 3:**
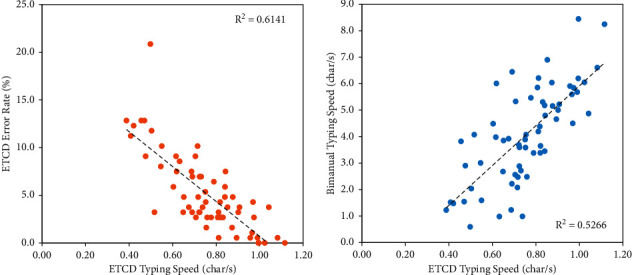
(a) Relationship between ETCD typing speed and error rate. (b) Relationship between ETCD typing speed and bimanual typing speed.

Figure 3(a) shows the relationship between ETCD typing speed and error rate. The error rate linearly decreased as typing speed increased with a Pearson correlation coefficient of 0.78.  Figure 3(b) shows the relationship between ETCD typing speed and bimanual typing speed. There was a linear relationship between the bimanual typing speed and ETCD typing speed with a Pearson correlation coefficient of 0.73. Highest values of ETCD typing speed corresponded to highest values of bimanual performance. On average, the bimanual performance was 5.3 times higher than the ETCD one (range: 1.2–9.7).

## 4. Discussion

The main purpose of this study was to assess the effect of age on eye-tracking performance of non-disabled adults using an ETCD for AAC. In addition, we compared eye-tracking performance with bimanual typing. We measured typing speed and error rate during the typing of a short text with a commercial eye-tracking device.

In the ETCD calibration phase, we encountered problems of calibration mainly in subjects with blue eyes, regardless of the light conditions of the room and/or of the angles and distance of the user from the camera. No specific dependence was observed with age. The rate of non-calibration that we obtained with our device (6.3%) was lower than that reported in other studies (about 9%) [22].

We initially stratified participants into six age decades, but based on the preliminary results we simplified the age stratification into three broader age groups. This grouping strategy is similar to that used in several neuropsychological measurements, e.g., reaction time measures [23–25].

### 4.1. Effect of Age on ETCD Performance

Our results in non-disabled individuals confirm that age has a negative impact on performance: as age increased, typing speed decreased and error rate increased. The fact that the aging process affects brain behavior, even in non-pathologic conditions, is well established. A similar aging effect was observed, for example, on execution time and error rate of eye-tracking data obtained during the evaluation of a project management software tool [26].

### 4.2. Learning Effect due to Trial Repetitions

Our protocol included 11 repetitions of the trial and hence of the associated visuomotor task, thus allowing assessment of the learning process typical of motor skill acquisition [27]. The typing speed performance of each age group (Figure 2) started at a different level (highest for the young and lowest for the elderly) and increased, with the largest improvements occurring in the first trials (experimental onset) and gradually leveling off. Similar behavior but with an exponential decaying trend was observed for the error rate. For both performance variables, the curves for the different age groups never intersected each other. The excellent *R*^2^ coefficient of the increasing/decaying models fitted over performance data testifies that the exponential model provides a very good representation of the learning process [28]. While the model's time constant for typing speed was roughly the same in the 3 age groups, the time constant for error rate for the elderly was about double that for the young and middle-aged groups. This was underlined also by the significant age ∗ group interaction observed in the two-way ANOVA analysis, and it could have implications for the development of a method for appropriate setup and implementation of ETCD (see below). As a rule, we could say that a stable performance could be obtained after 2 time constants when 86.5% of the maximum/minimum asymptotic value was reached. It is worth noting that the learning effect we observed in non-disabled subjects could represent a motivation reinforcement for AAC users using eye tracking for the first time, thus stimulating them to intensify practice.

### 4.3. Factors Influencing Error Rate

Concerning error rate performance, the results show that people with the lowest error rate were those who obtained the highest typing speed. This fact is confirmed by the strong correlation between these two variables [29]. This finding is in line with Dhakal et al. [30] who found that differences in performance were a result of differences in typing behavior. Although subjects improved their performance with learning due to the trial repetitions, their best performance did not involve a Fitts's law trade-off, by which fast movements (i.e., typing speed performance) should result in greater error rates [31]. This, likely because we did not provide any pressure to our participants, simply encouraged them to optimize their performance during experiments.

The error rate values we observed in this study are on average lower than those reported by another study that scored on average overproduction errors up to 28% [16]. A possible reason could be that the authors of that study used a keyboard with 8 large buttons (displayed in 2 rows with 4 columns) displaying letters that changed after each entry, based on a word prediction function with a preset internal dictionary. Conversely, in our study, we used a keyboard with a full layout showing all the alphabet letters always displayed in the same position and without the need to modify them in relation to the user's actions. This should constitute a support for motor memory in people undergoing a test. However, we note that this layout is quite different from the QWERTY layout typical of computer keyboards that could be more familiar for most users. Of course, the different layout could represent a potential confounding factor for our findings. On the other hand, the current older generation did not grow up with computer/on-screen keyboards and did not use a keyboard professionally, so they should have less experience than young people. The fact that all subjects were tested using a sequential (ABC) layout means that younger people also had to reorient themselves and search for the letters at first. In other words, the ABC layout could be considered a normalizing factor, thus putting all subjects in similar starting conditions.

### 4.4. Study Limitations

Our study has some limitations. First, the small sample size used (we enrolled about 10 subjects for each decade of age) lowers the statistical power by age decade, thus limiting the generalizability of our findings. For the provision of normative data, it is mandatory to increase the sample size and homogenize the distribution of education level, which was unbalanced in our sample toward a high level for young people and a low level for the elderly.

A second limitation is that in spite of the normalizing effect of the ABC layout, we cannot exclude that the low typing performance of the elderly could be partly due to the low typing frequency of this group. Possibly, future studies could address the effect on performance of different layouts and frequency of use.

Another limitation is that we used only one model of ETCD equipped with a virtual keyboard specifically designed to reduce overproduction errors. While this was done to maximize the technical performance, it prevents generalizing our findings to other virtual keyboards that often do not have this feature. However, absolute performance may not depend solely on the type of device and virtual keyboard used, so a deeper analysis of this question is warranted.

In the present study, we used only a 200 ms dwell-click time because this seemed to allow a better performance for most users, but the optimization of this parameter in disabled people affected by severe motor disease and communication problems needs to be verified with a dedicated study. Such study should ideally also use ETCDs with virtual keyboards incorporating an anti-Midas touch effect, which could require different optimization strategies.

### 4.5. Ideas for Future Research

The increase in availability of eye-tracking technology has fueled a sharp increase in implementations of interactive usage of eye tracking, and many people nowadays believe that gaze-based interaction may eventually become a standard human computer interface [19]. Given the lack of guidelines and directions regarding the proper setup of ETCD systems—other than the general rules proposed by ETCD producers [32] and the ISO standards on human-system interaction [33]—we propose here some criteria based on our findings which could be applied in future research to develop a method for ETCD setup and dwell time optimization.

When using eye-tracking devices for AAC, the most critical factor to set up is the dwell-click time because it has a direct influence on the typing speed and on overproduction errors due to the “Midas touch”[34, 35]. Various algorithmic methods have been proposed to correct the estimated gaze point, but many rely on post hoc processing that introduces high latency, making them unsuitable for real-time interaction [36]. In addition, there is no guarantee that the 200 ms dwell time suggested by Jacob et al. [12], which we adopted as a standard parameter in our study, is the optimal starting value in patients with severe motor diseases such as ALS.

Thus, we would suggest as a method for optimization to use a short text as we did in our study and start with a dwell-click time of 600 ms. This initial value can be reduced to 300–400 ms in the case of a young person. Following this, one should decrease the dwell-click time and repeat the test until a typing speed value is reached that is within the confidence limits provided in Table 2 for the age range in which the subject under test falls. When this happens, the test should be repeated without changing parameters at least 8–9 times for young and middle-aged people and 10–12 times for elderly people so as to verify minimization of the error rate. The number of proposed repetitions is related to the time constants of the exponential models fitted on our data. In particular, in young and middle-aged individuals, the typing speed time constant is prevalent over that of error rate. Conversely, for elderly people, the error rate time constant prevails over the typing speed time constant. It is estimated that excluding the time required to demonstrate functioning of the communication device, this procedure should take no more than 20–30 min, i.e., a time equivalent to that usually required for demonstration and learning.

The implementation of the proposed method requires, clearly, a dedicated validation study conducted in both disabled and non-disabled groups with the aim to verify if a learning effect similar to that of non-disabled subjects can be observed in people with progressive diseases and whether a progressive reduction of skills will impact also on learning time constants and performance.

## 5. Conclusions

Our results indicate that age has a negative impact on the ETCD performance of healthy subjects. In particular, with advancing age, the typing speed decreases and the error rate increases. Repetition of the evaluation protocol induces a learning effect, i.e., improved performance with an increase of typing speed and decrease in the error rate. This finding could represent a factor motivating practice for new AAC users. Based on these results, we propose for future investigation a method for the setup and tuning of eye-tracking devices for Augmentative and Alternative Communication.

## Figures and Tables

**Table 1:** Participant demographics.

	Age group	Subjects (*N*)	Sex	Dominant hand	Vision aid	Age (years) Mean (SD)	Education (years) Mean (SD)
Group 1	20–29	10	4M/6F	10R/0L	1G/1C/8N	24.1 (2.5)	15.7 (1.7)
Group 2	30–39	10	5M/5F	9R/1L	1G/3C/6N	35.2 (3.5)	17.5 (2.6)
Group 3	40–49	12	2M/10F	11R/1L	1G/3C/8N	44.6 (3.2)	13.6 (3.2)
Group 4	50–59	11	4M/7F	10R/1L	5G/0C/7N	54.9 (2.7)	17.0 (3.0)
Group 5	60–69	10	6M/4F	10R/0L	4G/0C/5N	63.4 (2.5)	13.9 (5.5)
Group 6	70–79	10	5M/5F	9R/1L	8G/0C/2N	74.7 (3.7)	8.0 (0.0)
		**63**	**26M/37F**	**59R/4L**	**20G/7C/36N**		

*N*, number; F, female; M, male; R, right; L, left; G, glasses; C, contact lenses; N, none.

**Table 2:** Typing speed and error rate across the 11 measurement sessions in the 3 age groups.

Variable	Age group		Session#											Mean	95% confidence limit
			1	2	3	4	5	6	7	8	9	10	11		
Typing speed	Young (*N* = 20)	Mean (char/s)	0.783	0.830	0.875	0.892	0.920	0.938	0.960	0.960	0.993	0.975	0.999	0.92	0.90–0.94
		SD	0.145	0.132	0.155	0.140	0.124	0.126	0.123	0.094	0.112	0.115	0.119		
	Middle-aged (*N* = 23)	Mean (char/s)	0.608	0.676	0.708	0.738	0.771	0.798	0.822	0.821	0.829	0.839	0.867	0.77	0.75–0.79
		SD	0.098	0.122	0.105	0.108	0.108	0.096	0.116	0.110	0.114	0.108	0.113		
	Elderly (*N* = 20)	Mean (char/s)	0.430	0.485	0.516	0.527	0.565	0.594	0.607	0.622	0.630	0.629	0.643	0.57	0.55–0.59
		SD	0.086	0.119	0.119	0.108	0.130	0.146	0.138	0.147	0.146	0.147	0.158		

Error rate	Young (*N* = 20)	Mean (%)	4.706	3.529	2.941	2.059	1.176	1.765	0.294	1.471	0.588	1.176	1.471	1.93	1.46–2.39
		SD	4.516	4.003	4.476	3.454	3.077	2.766	1.315	3.757	1.811	3.077	3.236		
	Middle-aged (*N* = 23)	Mean (%)	11.765	8.184	5.627	3.581	4.604	4.092	3.325	2.302	2.558	2.558	2.046	4.60	3.89–5.32
		SD	8.319	7.470	6.265	4.604	4.329	5.151	3.896	2.935	3.469	4.281	2.865		
	Elderly (*N* = 20)	Mean (%)	17.647	16.471	15.294	11.471	8.824	7.353	6.176	4.412	4.412	4.118	4.412	9.14	8.05–10.24
		SD	8.746	9.466	7.489	9.247	6.748	7.361	3.002	4.214	4.626	4.714	3.757		

**Table 3:** Exponential fitting features for ETCD typing speed and error rate in the three age groups.

	Age group	Time constant (# of sessions)	Adjusted *R*^2^
Typing speed	Young	4.67	0.96
	Middle-aged	4.54	0.99
	Elderly	4.98	0.99

Error rate	Young	2.35	0.86
	Middle-aged	2.02	0.96
	Elderly	5.84	0.96

## Data Availability

The data that support the findings of this study are available from the corresponding author upon reasonable request.

## Supplementary Materials

Supplementary Materials. Supplementary Material 1: Figure S1 shows the keyboard layout of the iAble device. Supplementary Material 2: (1) statistical analysis of demographic variables—63 subjects divided into 6 age groups; (2) preliminary ETCD typing speed analysis (6 age groups); (3) ETCD typing speed analysis (3 age groups); (4) ETCD error rate (3 age groups); (5) learning models; (6) relationship between ETCD typing speed and error rate; (7) relationship between ETCD typing speed and bimanual typing speed.
