# Supplementary material for: Writing with the Eyes: The Effect of Age on Eye-Tracking Performance in Non-Disabled Adults and a Comparison with Bimanual Typing
Source: Comput Intell Neurosci. 2021 Aug 24;2021:9365199. doi: 10.1155/2021/9365199 (PMC8410387; doi:10.1155/2021/9365199)

**Statistical Analysis**

**Statistical Methods**

Basic statistical analysis was performed on subjects’ demographic data and performance values. Data are expressed as frequency or mean value (±SD). We initially divided participants into 6 age groups (one for each decade) and performed a preliminary one-way ANOVA on ETCD typing speed with age group as the factor and Bonferroni test for pairwise comparisons. A significance level of < 0.05 was set for all the statistical tests. Based on the preliminary analysis, we collapsed the 6 age groups into 3 broader categories (Young, Middle-aged, and Elderly) and used them for the following analyses. Then, two-way repeated measures ANOVA with one between-subjects factor (age group) and one within-subjects factor (the repeated trials) was carried out on ETCD typing speed and error rate. In order to assess the differences between pairs of repeated sessions of each age group, one-way analysis and Bonferroni post-hoc test was executed for each age group category and session pair. In addition, using least square exponential fitting modelling, we assessed - for the same variables - the learning curve (i.e. the non-linear increase/decay) typical for each independent group to evaluate the minimum number of trials required to achieve a stable performance. For this, we considered that the learning was concluded (steady-state value reached) after a time corresponding to 2 time constants of the exponential function, i.e. when 86.5% of the maximum/minimum asymptotic value was reached. The increasing model y(x)=a-b*e^((- x/c)) and the decaying model y(x)=a+b*e^((- x/c) ) were fitted, respectively, on typing speed and error rate data.

Finally, correlation analysis was performed to assess the relationship between ETCD and bimanual typing performance variables.

Statistical analysis was performed using the STATA R13.0 statistical software package (StataCorp LLC, College Station, Texas, USA) and MATLAB custom software (2014b, The Math Works, Natick, MA, USA).

The following sections report full details of the statistical analysis.

1. **Demographic variables of the 63 subjects divided into 6 age groups**

Data are expressed as frequency or mean value (±SD)

|  | **Age group** | **Subjects (N)** | **Gender** | **Dominant hand** | **Vision aid** | **Mean age (years)** | **Mean education (years)** |
| --- | --- | --- | --- | --- | --- | --- | --- |
| **Group 1** | 20-29 | 10 | 4M/6F | 10R/0L | 1G/1C/8N | 24.1 (2.5) | 15.7 (1.7) |
| **Group 2** | 30-39 | 10 | 5M/5F | 9R/1L | 1G/3C/6N | 35.2 (3.5) | 17.5 (2.6) |
| **Group 3** | 40-49 | 12 | 10M/2F | 11R/1L | 1G/3C/8N | 44.6 (3.2) | 13,6 (3.2) |
| **Group 4** | 50-59 | 11 | 7M/4F | 10R/1L | 5G/0C/7N | 54.9 (2.7) | 17.0 (3.0) |
| **Group 5** | 60-69 | 10 | 4M/6F | 10R/0L | 4G/0C/5N | 63.4 (2.5) | 13.9 (5.5) |
| **Group 6** | 70-79 | 10 | 5M/5F | 9R/1L | 8G/0C/2N | 74.7 (3.7) | 8.0 (0.0) |
|  |  | **63** | **36M/27F** | **59R/4L** | **20G/7C/36N** |  |  |

1. **Preliminary ETCD Typing Speed analysis (6 age groups)**


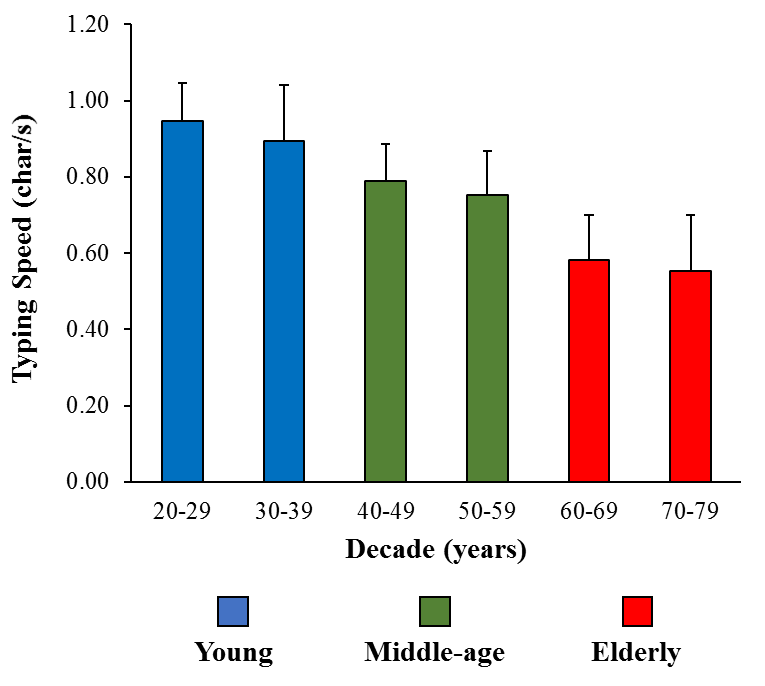


**Figure 1** ETCD mean typing speed and standard deviation for each age decade of the subjects who completed the study

Participants were divided into 6 age groups (one for each decade). Each decade was coded with a group number ranging from 1 to 6 and “TSMean” variable represents the tested data. Then a preliminary one-way ANOVA on ETCD data was performed.

The results of the one-way ANOVA are presented in the table below, with the F statistic and associated P value. A P value < 0.05, means that we can accept the hypothesis that there is an influence of the age group on the dependent variable (typing speed), and that at least two of the subgroups differ significantly. The Bartlett’s test was executed in order to assess homogeneity of variance.

***Stata Command: oneway TSMean Decade, bonferroni***

One Way Analysis of Variance

Source SS df MS F Prob > F

------------------------------------------------------------------------

Between groups 1.27962175 5 .255924351 23.06 0.0000

Within groups .632490049 57 .011096317

------------------------------------------------------------------------

Total 1.9121118 62 .030840513

Bartlett's test for equal variances: chi2(5) = 4.4184 Prob>chi2 = 0.491

The following table reports the Bonferroni multiple-comparison tests for each age subgroup pair.

**Bonferroni pairwise comparisons of TS-Mean by Decade: mean difference and p value**

Row Mean-|

Col Mean | 1 2 3 4 5

---------+-------------------------------------------------------

2 | -0.05

| 1.000

3 | -0.16 -0.11

| 0.013 0.332

4 | -0.20 -0.14 -0.04

| 0.001 0.044 1.000

5 | -0.36 -0.31 -0.21 -0.17

| 0.000 0.000 0.000 0.008

6 | -0.39 -0.34 -0.24 -0.20 -0.03

| 0.000 0.000 0.000 0.001 1.000

-------------------------------------------------------------------

Based on Oneway Anova analysis and Bonferroni test for all pairwise comparisons results, 3 different age groups (Young, Midlle-age, Elderly) were identified and reported with the same color in figure 1.

1. **ETCD Typing Speed analysis (3 age groups)**

Based on previous results, we collapsed the 6 age groups into Young, Middle-aged, and Elderly categories and used them for the following analyses.

**Table 2.** *Mean typing speed and SD across the 11 measurement sessions in the 3 age groups.*

| **Age Group** |  | **Session #** | | | | | | | | | | | Mean | 95% conf. lim. |
| --- | --- | --- | --- | --- | --- | --- | --- | --- | --- | --- | --- | --- | --- | --- |
|  |  | 1 | 2 | 3 | 4 | 5 | 6 | 7 | 8 | 9 | 10 | 11 |  |  |
| **Young**  **(N=20)** | Mean (char/s) | 0.783 | 0.830 | 0.875 | 0.892 | 0.920 | 0.938 | 0.960 | 0.960 | 0.993 | 0.975 | 0.999 | 0.92 | 0.90 - 0.94 |
|  | SD | 0.145 | 0.132 | 0.155 | 0.140 | 0.124 | 0.126 | 0.123 | 0.094 | 0.112 | 0.115 | 0.119 |  |  |
| **Middle-age**  **(N=23)** | Mean (char/s) | 0.608 | 0.676 | 0.708 | 0.738 | 0.771 | 0.798 | 0.822 | 0.821 | 0.829 | 0.839 | 0.867 | 0.77 | 0.75 - 0.79 |
|  | SD | 0.098 | 0.122 | 0.105 | 0.108 | 0.108 | 0.096 | 0.116 | 0.110 | 0.114 | 0.108 | 0.113 |  |  |
| **Elderly**  **(N=20)** | Mean (char/s) | 0.430 | 0.485 | 0.516 | 0.527 | 0.565 | 0.594 | 0.607 | 0.622 | 0.630 | 0.629 | 0.643 | 0.57 | 0.55 - 0.59 |
|  | SD | 0.086 | 0.119 | 0.119 | 0.108 | 0.130 | 0.146 | 0.138 | 0.147 | 0.146 | 0.147 | 0.158 |  |  |

*Cells in grey refer to pairwise comparisons significantly different from Session 1 values Abbreviations: N, number; SD, standard deviation.*

Two-way repeated measures ANOVA with one between-subjects factor (**AgeGrp**) and one within-subjects factor (the repeated trials: **Session**) was carried out on ETCD typing speed.

Three null hypotheses were tested in this procedure: a) factor AgeGrp does not influence the dependent variable TS (typing speed); b) factor Session does not influence variable TS and c) the effect of factor AgeGrp on variable TS does not depend on factor Session (i.e. there is no interaction of factors AgeGrp and Session).

If the calculated P-values for the two main effects a) and b), or for the 2-factor interaction was less than the conventional 0.05 (5%), then the corresponding null hypothesis was rejected, and the alternative hypothesis that there is indeed an influence must be accepted.

The results of the ANOVA are presented in the ANOVA table below, with the F statistic and associated P value.

**Two way ANOVA with one Between-subjects factor (AgeGrp) and one Within-subjects factor (Session)**

***Stata Command: anova TS AgeGrp / ID|AgeGrp Session AgeGrp#Session, repeated(Session)***

Number of obs = 693 R-squared = 0.9006

Root MSE = .067052 Adj R-squared = 0.8853

Source | Partial SS df MS F Prob > F

---------------+----------------------------------------------------

Model | 24.4356119 92 .265604477 59.08 0.0000

AgeGrp | 13.7910177 2 6.89550885 57.13 0.0000

ID|AgeGrp | 7.24221215 60 .120703536

---------------+----------------------------------------------------

Session | 3.32459306 10 .332459306 73.95 0.0000

AgeGrp#Session | .03230769 20 .001615384 0.36 0.9958

Residual | 2.69761805 600 .00449603

---------------+----------------------------------------------------

Total | 27.1332299 692 .03920987

Between-subjects error term: ID|AgeGrp

Levels: 63 (60 df)

Lowest b.s.e. variable: ID

Covariance pooled over: AgeGrp (for repeated variable)

Repeated variable: Session

Huynh-Feldt epsilon = 0.6257

Greenhouse-Geisser epsilon = 0.5450

Box's conservative epsilon = 0.1000

------------ Prob > F ------------

Source | df F Regular H-F G-G Box

---------------+----------------------------------------------------

Session | 10 73.95 0.0000 0.0000 0.0000 0.0000

AgeGrp#Session | 20 0.36 0.9958 0.9789 0.9698 0.6997

Residual | 600

--------------------------------------------------------------------

In order to assess typing speed (TS) differences between different sessions of each age group, one-way analysis and Bonferroni post-hoc test was executed for each AgeGrp value.

The results of the ANOVA are presented in the ANOVA table below, with the F statistic and associated P value. The following table reports the Bonferroni multiple-comparison tests for each age subgroup and mean difference and p values are reported for each session pair.

***Stata Command: by AgeGrp, sort : oneway TS Session, bonferroni tabulate nomeans nostandard nofreq wrap***

-> AgeGrp = 1

Analysis of Variance

Source SS df MS F Prob > F

------------------------------------------------------------------------

Between groups .957571695 10 .09575717 5.95 0.0000

Within groups 3.36314342 209 .016091595

------------------------------------------------------------------------

Total 4.32071512 219 .019729293

Bartlett's test for equal variances: chi2(10) = 6.7301 Prob>chi2 = 0.751

Comparison of TS by Session (Bonferroni)

Row Mean-|

Col Mean | 1 2 3 4 5 6 7 8 9 10

---------+--------------------------------------------------------------------------------------------------------------

2 | 0.05

| 1.000

3 | 0.09 0.05

| 1.000 1.000

4 | 0.11 0.06 0.02

| 0.385 1.000 1.000

5 | 0.14 0.09 0.05 0.03

| 0.041 1.000 1.000 1.000

6 | 0.16 0.11 0.06 0.05 0.02

| 0.008 0.399 1.000 1.000 1.000

7 | 0.18 0.13 0.08 0.07 0.04 0.02

| 0.001 0.074 1.000 1.000 1.000 1.000

8 | 0.18 0.13 0.08 0.07 0.04 0.02 0.00

| 0.001 0.074 1.000 1.000 1.000 1.000 1.000

9 | 0.21 0.16 0.12 0.10 0.07 0.05 0.03 0.03

| 0.000 0.004 0.197 0.699 1.000 1.000 1.000 1.000

10 | 0.19 0.15 0.10 0.08 0.05 0.04 0.01 0.01 -0.02

| 0.000 0.021 0.758 1.000 1.000 1.000 1.000 1.000 1.000

11 | 0.22 0.17 0.12 0.11 0.08 0.06 0.04 0.04 0.01 0.02

| 0.000 0.002 0.121 0.453 1.000 1.000 1.000 1.000 1.000 1.000

---------------------------------------------------------------------------------------------------------------------------

-> AgeGrp = 2

Analysis of Variance

Source SS df MS F Prob > F

------------------------------------------------------------------------

Between groups 1.47335003 10 .147335003 12.34 0.0000

Within groups 2.89037276 242 .011943689

------------------------------------------------------------------------

Total 4.36372279 252 .01731636

Bartlett's test for equal variances: chi2(10) = 1.9872 Prob>chi2 = 0.996

Comparison of TS by Session (Bonferroni)

Row Mean-|

Col Mean | 1 2 3 4 5 6 7 8 9 10

---------+--------------------------------------------------------------------------------------------------------------

2 | 0.07

| 1.000

3 | 0.10 0.03

| 0.116 1.000

4 | 0.13 0.06 0.03

| 0.004 1.000 1.000

5 | 0.16 0.10 0.06 0.03

| 0.000 0.187 1.000 1.000

6 | 0.19 0.12 0.09 0.06 0.03

| 0.000 0.010 0.299 1.000 1.000

7 | 0.21 0.15 0.11 0.08 0.05 0.02

| 0.000 0.000 0.025 0.507 1.000 1.000

8 | 0.21 0.14 0.11 0.08 0.05 0.02 -0.00

| 0.000 0.001 0.030 0.587 1.000 1.000 1.000

9 | 0.22 0.15 0.12 0.09 0.06 0.03 0.01 0.01

| 0.000 0.000 0.011 0.262 1.000 1.000 1.000 1.000

10 | 0.23 0.16 0.13 0.10 0.07 0.04 0.02 0.02 0.01

| 0.000 0.000 0.003 0.101 1.000 1.000 1.000 1.000 1.000

11 | 0.26 0.19 0.16 0.13 0.10 0.07 0.05 0.05 0.04 0.03

| 0.000 0.000 0.000 0.004 0.172 1.000 1.000 1.000 1.000 1.000

----------------------------------------------------------------------------------------------------------------------------

-> AgeGrp = 3

Analysis of Variance

Source SS df MS F Prob > F

------------------------------------------------------------------------

Between groups .971460288 10 .097146029 5.51 0.0000

Within groups 3.68631401 209 .017637866

------------------------------------------------------------------------

Total 4.6577743 219 .021268376

Bartlett's test for equal variances: chi2(10) = 10.4841 Prob>chi2 = 0.399

Comparison of TS by Session (Bonferroni)

Row Mean-|

Col Mean | 1 2 3 4 5 6 7 8 9 10

---------+--------------------------------------------------------------------------------------------------------------

2 | 0.05

| 1.000

3 | 0.09 0.03

| 1.000 1.000

4 | 0.10 0.04 0.01

| 1.000 1.000 1.000

5 | 0.13 0.08 0.05 0.04

| 0.088 1.000 1.000 1.000

6 | 0.16 0.11 0.08 0.07 0.03

| 0.007 0.547 1.000 1.000 1.000

7 | 0.18 0.12 0.09 0.08 0.04 0.01

| 0.002 0.221 1.000 1.000 1.000 1.000

8 | 0.19 0.14 0.11 0.10 0.06 0.03 0.02

| 0.000 0.070 0.654 1.000 1.000 1.000 1.000

9 | 0.20 0.14 0.11 0.10 0.07 0.04 0.02 0.01

| 0.000 0.038 0.392 0.842 1.000 1.000 1.000 1.000

10 | 0.20 0.14 0.11 0.10 0.06 0.04 0.02 0.01 -0.00

| 0.000 0.039 0.404 0.867 1.000 1.000 1.000 1.000 1.000

11 | 0.21 0.16 0.13 0.12 0.08 0.05 0.04 0.02 0.01 0.01

| 0.000 0.012 0.151 0.348 1.000 1.000 1.000 1.000 1.000 1.000 -------------------------------------------------------------------------------------------------------------------------

Summary Table for Typing Speed pairwise comparisons for each age group

| **Age Group** | **Session** | **Mean** | **Different (P<0.05)**  **from Session nr** |
| --- | --- | --- | --- |
| Young  (N=20) | 1 | 0.7831 | 5,6,7,8,9,10,11 |
|  | 2 | 0.8296 | 9,10,11 |
|  | 3 | 0.8751 |  |
|  | 4 | 0.8924 |  |
|  | 5 | 0.9205 | 1 |
|  | 6 | 0.9384 | 1 |
|  | 7 | 0.96 | 1 |
|  | 8 | 0.96 | 1 |
|  | 9 | 0.9933 | 1,2 |
|  | 10 | 0.9747 | 1,2 |
|  | 11 | 0.9994 | 1,2 |
| Middle-age  (N=23) | 1 | 0.6076 | 4,5,6,7,8,9,10,11 |
|  | 2 | 0.6758 | 6,7,8,9,10,11 |
|  | 3 | 0.7078 | 7,8,9,10,11 |
|  | 4 | 0.7377 | 11 |
|  | 5 | 0.7711 | 1 |
|  | 6 | 0.7982 | 1,2 |
|  | 7 | 0.8223 | 1,2,3 |
|  | 8 | 0.8206 | 1,2,3 |
|  | 9 | 0.8295 | 1,2,3 |
|  | 10 | 0.8392 | 1,2,3 |
|  | 11 | 0.8673 | 1,2,3,4 |
| Elderly  (N=20) | 1 | 0.4304 | 6,7,8,9,10,11 |
|  | 2 | 0.4849 | 9,10,11 |
|  | 3 | 0.5155 |  |
|  | 4 | 0.527 |  |
|  | 5 | 0.5646 |  |
|  | 6 | 0.5942 | 1 |
|  | 7 | 0.6071 | 1 |
|  | 8 | 0.6221 | 1 |
|  | 9 | 0.6297 | 1,2 |
|  | 10 | 0.6292 | 1,2 |
|  | 11 | 0.6428 | 1,2 |

1. **ETCD Error rate (3 age groups)**

The ETCD error rate variable was assessed through statistical analysis similar to that reported above for the typing speed variable.

**Table 3.** *Mean error rate and SD across the 11 measurement sessions in the 3 age groups.*

| **Age Group** |  | **Session #** | | | | | | | | | | | Mean | 95% conf. lim. |
| --- | --- | --- | --- | --- | --- | --- | --- | --- | --- | --- | --- | --- | --- | --- |
|  |  | 1 | 2 | 3 | 4 | 5 | 6 | 7 | 8 | 9 | 10 | 11 |  |  |
| **Young**  **(N=20)** | Mean (char/s) | 4.706 | 3.529 | 2.941 | 2.059 | 1.176 | 1.765 | 0.294 | 1.471 | 0.588 | 1.176 | 1.471 | 1.93 | 1.46 - 2.39 |
|  | SD | 4.516 | 4.003 | 4.476 | 3.454 | 3.077 | 2.766 | 1.315 | 3.757 | 1.811 | 3.077 | 3.236 |  |  |
| **Middle-age**  **(N=23)** | Mean (char/s) | 11.765 | 8.184 | 5.627 | 3.581 | 4.604 | 4.092 | 3.325 | 2.302 | 2.558 | 2.558 | 2.046 | 4.60 | 3.89 - 5.32 |
|  | SD | 8.319 | 7.470 | 6.265 | 4.604 | 4.329 | 5.151 | 3.896 | 2.935 | 3.469 | 4.281 | 2.865 |  |  |
| **Elderly**  **(N=20)** | Mean (char/s) | 17.647 | 16.471 | 15.294 | 11.471 | 8.824 | 7.353 | 6.176 | 4.412 | 4.412 | 4.118 | 4.412 | 9.14 | 8.05 - 10.24 |
|  | SD | 8.746 | 9.466 | 7.489 | 9.247 | 6.748 | 7.361 | 3.002 | 4.214 | 4.626 | 4.714 | 3.757 |  |  |

***Stata Command: anova ER AgeGrp / ID|AgeGrp Session AgeGrp#Session, repeated(Session)***

Number of obs = 693 R-squared = 0.6063

Root MSE = 4.57439 Adj R-squared = 0.5459

Source | Partial SS df MS F Prob > F

---------------+----------------------------------------------------

Model | 19331.8947 92 210.12929 10.04 0.0000

|

AgeGrp | 5872.22045 2 2936.11023 31.43 0.0000

ID|AgeGrp | 5605.78251 60 93.4297085

---------------+----------------------------------------------------

Session | 5929.19174 10 592.919174 28.34 0.0000

AgeGrp#Session | 1940.48178 20 97.024089 4.64 0.0000

|

Residual | 12555.0419 600 20.9250699

---------------+----------------------------------------------------

Total | 31886.9366 692 46.0793881

Between-subjects error term: ID|AgeGrp

Levels: 63 (60 df)

Lowest b.s.e. variable: ID

Covariance pooled over: AgeGrp (for repeated variable)

Repeated variable: Session

Huynh-Feldt epsilon = 0.7049

Greenhouse-Geisser epsilon = 0.6067

Box's conservative epsilon = 0.1000

------------ Prob > F ------------

Source | df F Regular H-F G-G Box

---------------+----------------------------------------------------

Session | 10 28.34 0.0000 0.0000 0.0000 0.0000

AgeGrp#Session | 20 4.64 0.0000 0.0000 0.0000 0.0134

Residual | 600

--------------------------------------------------------------------

One-Way analysis and Bonferroni post-hoc test by AgeGrp on ETCD error rate

***Stata Command: by AgeGrp, sort : oneway ER Session, bonferroni tabulate nomeans nostandard nofreq wrap***

-> AgeGrp = 1

Analysis of Variance

Source SS df MS F Prob > F

------------------------------------------------------------------------

Between groups 347.279019 10 34.7279019 3.07 0.0012

Within groups 2366.78201 209 11.3243158

------------------------------------------------------------------------

Total 2714.06103 219 12.3929727

Bartlett's test for equal variances: chi2(10) = 40.4266 Prob>chi2 = 0.000

Comparison of ER by Session (Bonferroni)

Row Mean-|

Col Mean | 1 2 3 4 5 6 7 8 9 10

---------+--------------------------------------------------------------------------------------------------------------

2 | -1.17647

| 1.000

|

3 | -1.76471 -.588235

| 1.000 1.000

|

4 | -2.64706 -1.47059 -.882353

| 0.751 1.000 1.000

|

5 | -3.52941 -2.35294 -1.76471 -.882353

| 0.059 1.000 1.000 1.000

|

6 | -2.94118 -1.76471 -1.17647 -.294118 .588235

| 0.342 1.000 1.000 1.000 1.000

|

7 | -4.41176 -3.23529 -2.64706 -1.76471 -.882353 -1.47059

| 0.003 0.147 0.751 1.000 1.000 1.000

|

8 | -3.23529 -2.05882 -1.47059 -.588235 .294118 -.294118 1.17647

| 0.147 1.000 1.000 1.000 1.000 1.000 1.000

|

9 | -4.11765 -2.94118 -2.35294 -1.47059 -.588235 -1.17647 .294118 -.882353

| 0.008 0.342 1.000 1.000 1.000 1.000 1.000 1.000

|

10 | -3.52941 -2.35294 -1.76471 -.882353 0 -.588235 .882353 -.294118 .588235

| 0.059 1.000 1.000 1.000 1.000 1.000 1.000 1.000 1.000

|

11 | -3.23529 -2.05882 -1.47059 -.588235 .294118 -.294118 1.17647 0 .882353 .294118

| 0.147 1.000 1.000 1.000 1.000 1.000 1.000 1.000 1.000 1.000

----------------------------------------------------------------------------------------------------------------------------------------

-> AgeGrp = 2

Analysis of Variance

Source SS df MS F Prob > F

------------------------------------------------------------------------

Between groups 2030.99142 10 203.099142 7.62 0.0000

Within groups 6448.02166 242 26.6447176

------------------------------------------------------------------------

Total 8479.01309 252 33.6468773

Bartlett's test for equal variances: chi2(10) = 55.0146 Prob>chi2 = 0.000

Comparison of ER by Session

(Bonferroni)

Row Mean-|

Col Mean | 1 2 3 4 5 6 7 8 9 10

---------+--------------------------------------------------------------------------------------------------------------

2 | -3.58056

| 1.000

|

3 | -6.13811 -2.55754

| 0.004 1.000

|

4 | -8.18414 -4.60358 -2.04604

| 0.000 0.152 1.000

|

5 | -7.16113 -3.58056 -1.02302 1.02302

| 0.000 1.000 1.000 1.000

|

6 | -7.67263 -4.09207 -1.53453 .511509 -.511509

| 0.000 0.422 1.000 1.000 1.000

|

7 | -8.4399 -4.85934 -2.30179 -.255754 -1.27877 -.767263

| 0.000 0.088 1.000 1.000 1.000 1.000

|

8 | -9.46292 -5.88235 -3.32481 -1.27877 -2.30179 -1.79028 -1.02302

| 0.000 0.008 1.000 1.000 1.000 1.000 1.000

|

9 | -9.20716 -5.6266 -3.06905 -1.02302 -2.04604 -1.53453 -.767263 .255754

| 0.000 0.015 1.000 1.000 1.000 1.000 1.000 1.000

|

10 | -9.20716 -5.6266 -3.06905 -1.02302 -2.04604 -1.53453 -.767263 .255754 0

| 0.000 0.015 1.000 1.000 1.000 1.000 1.000 1.000 1.000

|

11 | -9.71867 -6.13811 -3.58056 -1.53453 -2.55754 -2.04604 -1.27877 -.255754 -.511509 -.511509

| 0.000 0.004 1.000 1.000 1.000 1.000 1.000 1.000 1.000 1.000

----------------------------------------------------------------------------------------------------------------------------------------

-> AgeGrp = 3

Analysis of Variance

Source SS df MS F Prob > F

------------------------------------------------------------------------

Between groups 5475.62126 10 547.562126 12.24 0.0000

Within groups 9346.02076 209 44.7178027

------------------------------------------------------------------------

Total 14821.642 219 67.6787307

Bartlett's test for equal variances: chi2(10) = 52.1704 Prob>chi2 = 0.000

Comparison of ER by Session

(Bonferroni)

Row Mean-|

Col Mean | 1 2 3 4 5 6 7 8 9 10

---------+--------------------------------------------------------------------------------------------------------------

2 | -1.17647

| 1.000

|

3 | -2.35294 -1.17647

| 1.000 1.000

|

4 | -6.17647 -5 -3.82353

| 0.213 1.000 1.000

|

5 | -8.82353 -7.64706 -6.47059 -2.64706

| 0.002 0.021 0.138 1.000

|

6 | -10.2941 -9.11765 -7.94118 -4.11765 -1.47059

| 0.000 0.001 0.012 1.000 1.000

|

7 | -11.4706 -10.2941 -9.11765 -5.29412 -2.64706 -1.17647

| 0.000 0.000 0.001 0.718 1.000 1.000

|

8 | -13.2353 -12.0588 -10.8824 -7.05882 -4.41176 -2.94118 -1.76471

| 0.000 0.000 0.000 0.055 1.000 1.000 1.000

|

9 | -13.2353 -12.0588 -10.8824 -7.05882 -4.41176 -2.94118 -1.76471 0

| 0.000 0.000 0.000 0.055 1.000 1.000 1.000 1.000

|

10 | -13.5294 -12.3529 -11.1765 -7.35294 -4.70588 -3.23529 -2.05882 -.294118 -.294118

| 0.000 0.000 0.000 0.034 1.000 1.000 1.000 1.000 1.000

|

11 | -13.2353 -12.0588 -10.8824 -7.05882 -4.41176 -2.94118 -1.76471 0 0 .294118

| 0.000 0.000 0.000 0.055 1.000 1.000 1.000 1.000 1.000 1.000

Summary Table for ETCD error rate pairwise comparisons for each age group

| **Age Group** | **Session** | **Mean**  **(%)** | **Different (P<0.05)**  **from Session nr** |
| --- | --- | --- | --- |
| Young  (N=20) | 1 | 4.71 | 7, 9 |
|  | 2 | 3.53 |  |
|  | 3 | 2.94 |  |
|  | 4 | 2.06 |  |
|  | 5 | 1.18 |  |
|  | 6 | 1.76 |  |
|  | 7 | 0.29 | 1 |
|  | 8 | 1.47 |  |
|  | 9 | 0.59 | 1 |
|  | 10 | 1.18 |  |
|  | 11 | 1.47 |  |
| Middle-age  (N=23) | 1 | 11.76 | 3, 4, 5, 6, 7, 8, 9, 10, 11 |
|  | 2 | 8.18 | 8, 9, 10, 11 |
|  | 3 | 5.63 | 1 |
|  | 4 | 3.58 | 1 |
|  | 5 | 4.60 | 1 |
|  | 6 | 4.09 | 1 |
|  | 7 | 3.32 | 1 |
|  | 8 | 2.30 | 1, 2 |
|  | 9 | 2.56 | 1, 2 |
|  | 10 | 2.56 | 1, 2 |
|  | 11 | 2.05 | 1, 2 |
| Elderly  (N=20) | 1 | 17.65 | 5, 6, 7, 8, 9, 10, 11 |
|  | 2 | 16.47 | 5, 6, 7, 8, 9, 10, 11 |
|  | 3 | 15.29 | 6, 7, 8, 9, 10, 11 |
|  | 4 | 11.47 | 10 |
|  | 5 | 8.82 | 1, 2 |
|  | 6 | 7.35 | 1, 2, 3 |
|  | 7 | 6.18 | 1, 2, 3 |
|  | 8 | 4.41 | 1, 2, 3 |
|  | 9 | 4.41 | 1, 2, 3 |
|  | 10 | 4.12 | 1, 2, 3, 4 |
|  | 11 | 4.41 | 1, 2, 3 |

1. **Learning Models**

Learning models fitted to describe the learning phenomenon due to repeated measurement protocol was assessed by using nonlinear least square exponential fitting method. In particular we assessed on both performance variables the learning curve (i.e. the non-linear increase /decay) typical for each independent group to evaluate the minimum number of trials required to achieve a stable performance.

The increasing model y(x)=a-b*e^((- x/c)) and the decaying model y(x)=a+b*e^((- x/c) ), were fitted respectively, on typing speed and error rate data. Exponentials are often used when the rate of change of a quantity is proportional to the initial amount of the quantity. If the coefficient associated with b is negative, y represents exponential decay. If the coefficient is positive, y represents exponential growth. In the above reported formulas, y is the dependent (fitted) variable (i.e. typing speed and error rate) and x is the session number.

For the model we implemented, the learning was considered concluded when a steady-state value was reached after a time corresponding to 2 time constants, i.e. the coefficient of the exponential function ( i.e. when 86.5% of the maximum/minimum asymptotic value was reached).


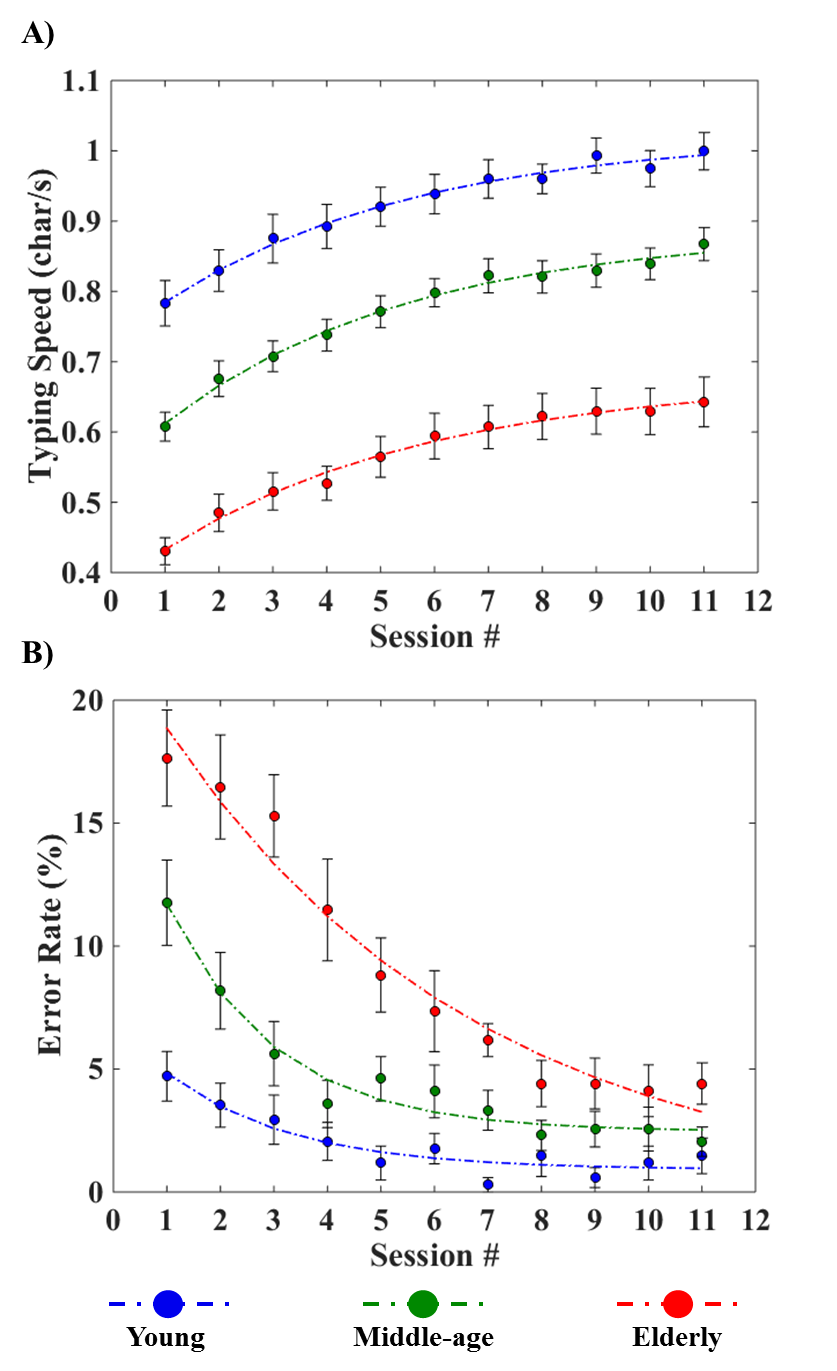


**Figure 2.** *A) Mean typing speed with standard error and B) Mean error rate with standard error across the 11 measurement sessions in the 3 age groups. Dashed lines represent the increasing/decaying exponential model fitted on the measured data. Error bars represent the standard error of the mean in each measurement session and age group.*

The tables below report for each variable and age group the model, the fitted coefficients, their 95% confidence limits and the results of the goodness of fit analysis measuring the discrepancy between the observed values and those that would be expected of the model in a normal distribution case.

**Fitting results**

| **Typing Speed** | Fit results | Goodness of fit |
| --- | --- | --- |
| Young | General model:  fitresult(x) = a-b*exp(-x/c)  Coefficients (with 95% confidence bounds):  a = 1.022 (0.9884, 1.055)  b = 0.2939 (0.2671, 0.3208)  c = 4.666 (3.078, 6.255) | sse: 5.7274e-04  rsquare: 0.9880  dfe: 8  adjrsquare: 0.9850  rmse: 0.0085 |
| Middle-age | General model:  fitresult(x) = a-b*exp(-x/c)  Coefficients (with 95% confidence bounds):  a = 0.885 (0.8521, 0.9179)  b = 0.34 (0.3126, 0.3674)  c = 4.543 (3.193, 5.893) | sse: 6.0944e-04  rsquare: 0.9905  dfe: 8  adjrsquare: 0.9881  rmse: 0.0087 |
| Elderly | General model:  fitresult(x) = a-b*exp(-x/c)  Coefficients (with 95% confidence bounds):  a = 0.6764 (0.6419, 0.7109)  b = 0.2978 (0.2717, 0.3239)  c = 4.984 (3.351, 6.618) | sse: 4.8699e-04  rsquare: 0.9900  dfe: 8  adjrsquare: 0.9875  rmse: 0.0078 |

| **Error Rate** | Fit results | Goodness of fit |
| --- | --- | --- |
| Young | General model:  fitresult(x) = a+b*exp(-x/c)  Coefficients (with 95% confidence bounds):  a = 0.9079 (0.1707, 1.645)  b = 6.002 (3.455, 8.55)  c = 2.346 (0.6067, 4.085) | sse: 1.9716  rsquare: 0.8865  dfe: 8  adjrsquare: 0.8581  rmse: 0.4964 |
| Middle-age | General model:  fitresult(x) = a+b*exp(-x/c)  Coefficients (with 95% confidence bounds):  a = 2.46 (1.657, 3.263)  b = 15.12 (11.35, 18.9)  c = 2.025 (1.26, 2.79) | sse: 3.1009  rsquare: 0.9649  dfe: 8  adjrsquare: 0.9561  rmse: 0.6226 |
| Elderly | General model:  fitresult(x) = a+b*exp(-x/c)  Coefficients (with 95% confidence bounds):  a = -0.182 (-6.535, 6.171)  b = 22.59 (17.97, 27.22)  c = 5.841 (1.764, 9.917) | sse: 9.3734  rsquare: 0.9658  dfe: 8  adjrsquare: 0.9572  rmse: 1.0824 |

**Table 4.** Exponential fitting model features for typing speed and error rate in the three age groups

|  | **Age Group** | **Time constant**  **(# of sessions)** | **Adjusted R^2^** |
| --- | --- | --- | --- |
| **Typing speed** | Young | 4.67 | 0.96 |
|  | Middle-age | 4.54 | 0.99 |
|  | Elderly | 4.98 | 0.99 |
| **Error Rate** | Young | 2.35 | 0.86 |
|  | Middle-age | 2.02 | 0.96 |
|  | Elderly | 5.84 | 0.96 |

1. **Relationship between ETCD Typing Speed and Error Rate** **carried out by correlation analysis implemented through linear regression.**


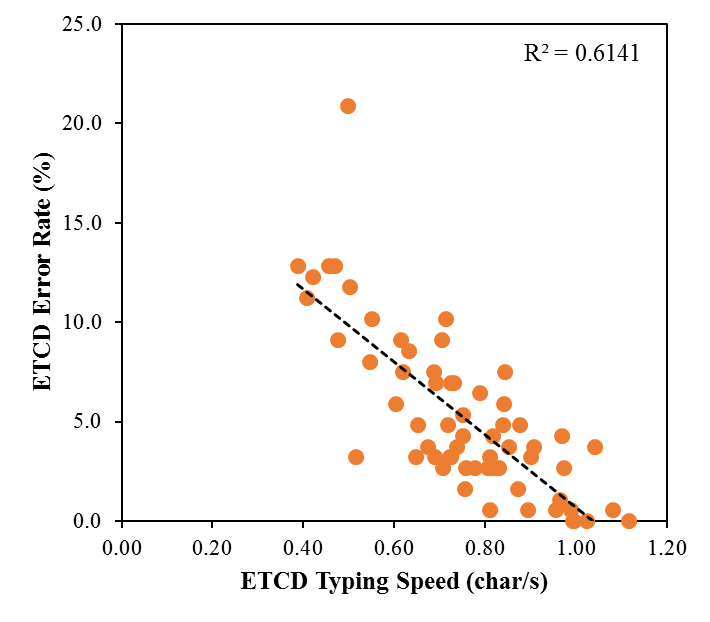


1. **Relationship between ETCD Typing Speed and Bimanual Typing Speed carried out by correlation analysis implemented through linear regression.**


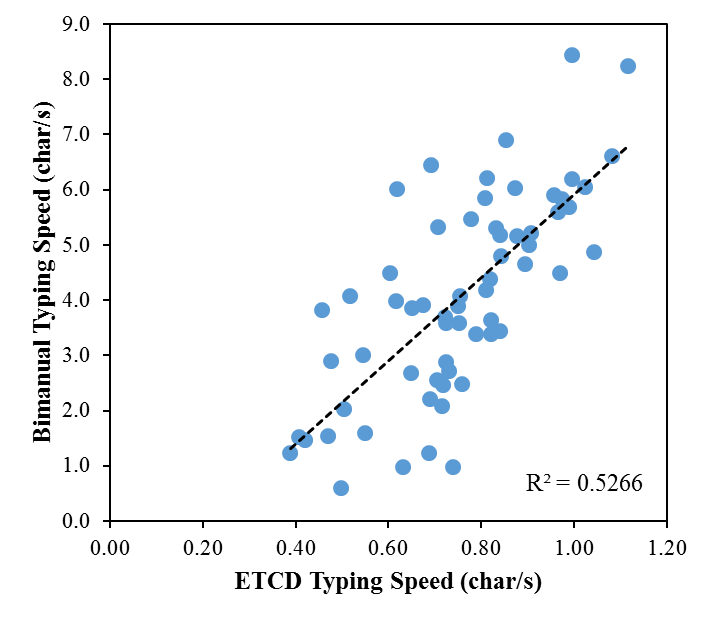

Supplement: Supplementary Materials. — Supplementary Material 1: Figure S1 shows the keyboard layout of the iAble device. Supplementary Material 2: (1) statistical analysis of demographic variables—63 subjects divided into 6 age groups; (2) preliminary ETCD typing speed analysis (6 age groups); (3) ETCD typing speed analysis (3 age groups); (4) ETCD error rate (3 age groups); (5) learning models; (6) relationship between ETCD typing speed and error rate; (7) relationship between ETCD typing speed and bimanual typing speed. [file 9365199.f1.zip › 9365199.f1/Supplementary Material 2 (1).docx]
