# Supplementary figures and images for: Writing with the Eyes: The Effect of Age on Eye-Tracking Performance in Non-Disabled Adults and a Comparison with Bimanual Typing
Source: Comput Intell Neurosci. 2021 Aug 24;2021:9365199. doi: 10.1155/2021/9365199 (PMC8410387; doi:10.1155/2021/9365199)

**Figure S1.** Keyboard layout of the iAble device.


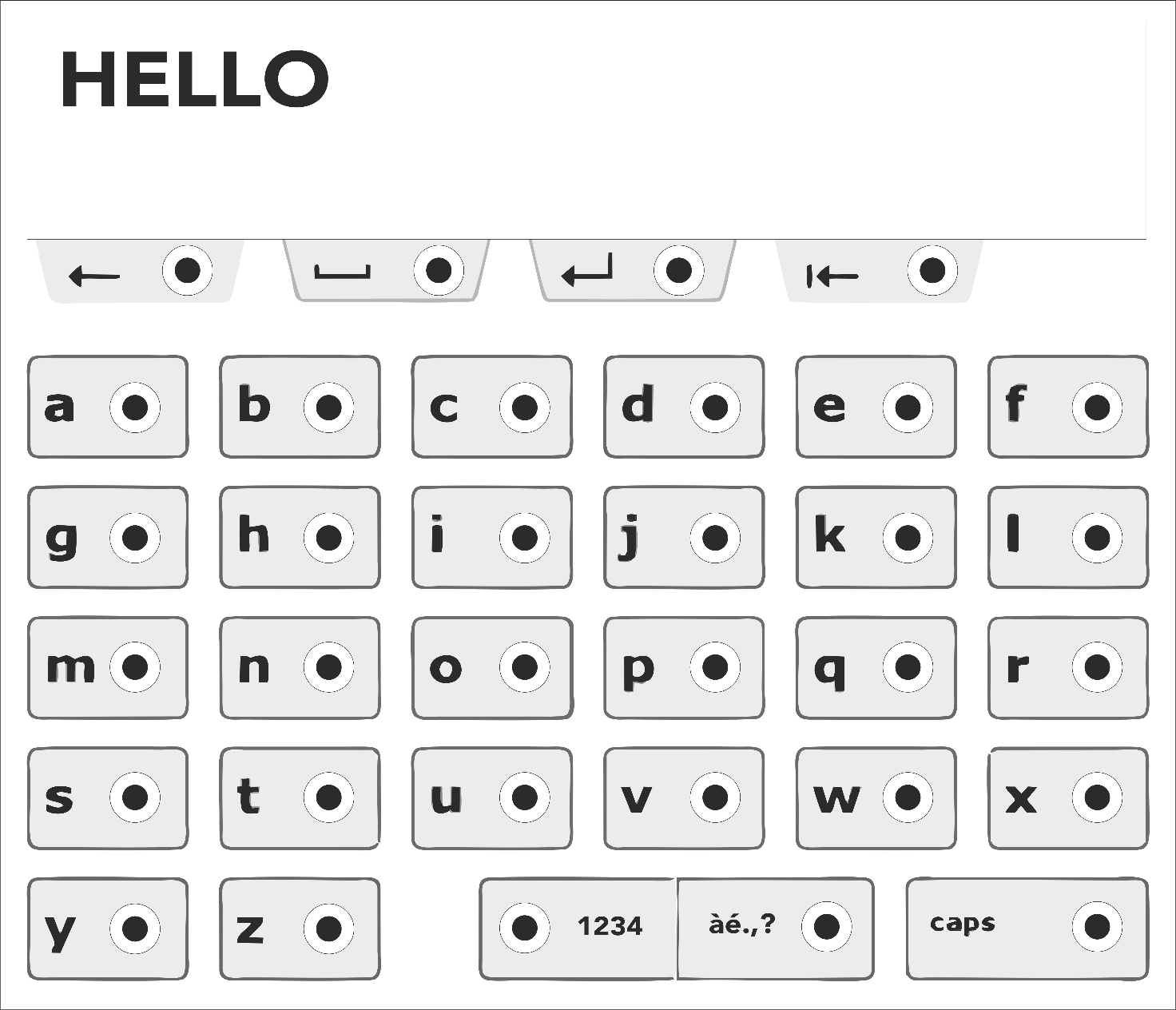

Supplement: Supplementary Materials. — Supplementary Material 1: Figure S1 shows the keyboard layout of the iAble device. Supplementary Material 2: (1) statistical analysis of demographic variables—63 subjects divided into 6 age groups; (2) preliminary ETCD typing speed analysis (6 age groups); (3) ETCD typing speed analysis (3 age groups); (4) ETCD error rate (3 age groups); (5) learning models; (6) relationship between ETCD typing speed and error rate; (7) relationship between ETCD typing speed and bimanual typing speed. [file 9365199.f1.zip › 9365199.f1/Supplementary Material 1.docx]
